# Exploring anesthetic-induced gene expression changes and immune cell dynamics in atrial tissue post-coronary artery bypass graft surgery

**DOI:** 10.1515/med-2024-1014

**Published:** 2024-08-13

**Authors:** Mengmeng Bao, Anshi Wu

**Affiliations:** Department of Anesthesiology, Beijing Chaoyang Hospital, Capital Medical University, Beijing, 100020, China

**Keywords:** anesthetic modulation, gene expression profiling, immune cell infiltration, coronary artery bypass graft, atrial tissue analysis

## Abstract

**Background:**

This study leverages the GSE4386 dataset, obtained from atrial tissue samples post-coronary artery bypass graft (CABG) surgery, to investigate the impact of anesthetic agents (sevoflurane and propofol) on gene expression and immune cell infiltration.

**Methods:**

Hierarchical clustering and box plots were employed for dataset preprocessing, highlighting a significant outlier (sample GSM99282), subsequently removed to ensure data integrity. Differentially expressed genes (DEGs) were identified using volcano plots based on specific log-fold-change and *P*-value thresholds. Additional analyses included the Friends approach, Spearman’s correlation, and gene set enrichment analysis (GSEA), exploring functional annotations and pathways.

**Results:**

Heatmaps and bubble plots depicted DEGs, revealing distinct expression patterns between the sevoflurane and propofol groups. Friends analysis identified top genes based on log fold changes, further correlated using Spearman’s method. Gene Ontology and Kyoto Encyclopedia of Genes and Genomes enrichment analyses illustrated functional annotations of DEGs, while GSEA highlighted enriched biological categories. Immune cell infiltration analysis showcased varied cellular presence post-CABG. ESTIMATE algorithm scores demonstrated differences in immune, stroma, and estimate scores. Microenvironment Cell Populations-counter (MCPcounter) revealed an increased abundance of cytotoxic lymphocytes in the sevoflurane group, confirmed by a single sample GSEA. CIBERSORT algorithm identified distinct immune cell compositions, highlighting differences in macrophage M0 prevalence between sevoflurane and propofol groups.

**Conclusions:**

This comprehensive analysis provides insights into anesthetic-induced gene expression changes and immune cell dynamics in atrial tissue post-CABG surgery. The identified DEGs and immune cell compositions offer potential biomarkers and therapeutic targets for refining anesthetic strategies in cardiac surgeries.

## 1 Introduction

Cardiovascular diseases remain a leading cause of morbidity and mortality globally, necessitating intricate surgical interventions such as CABG surgeries [1]. The success of these procedures relies not only on surgical precision but also on the management of perioperative factors, including anesthesia [2,3]. Anesthesia, particularly the use of agents like sevoflurane and propofol [4,5,6], plays a pivotal role in ensuring patient comfort and procedural efficacy. However, the molecular and immunological consequences of these anesthetic agents on atrial tissues post-CABG surgery are not fully understood. This study delves into the intricate interplay between anesthetic-induced gene expression changes and immune cell dynamics within atrial tissue, employing a comprehensive analysis of the GSE4386 dataset derived from post-CABG surgery samples.

The significance of investigating anesthetic-induced gene expression changes and immune cell dynamics lies in the potential implications for patient recovery and long-term cardiovascular health [7,8]. The molecular responses within atrial tissues post-CABG can provide valuable insights into the mechanisms underlying anesthesia-associated effects, paving the way for personalized and optimized anesthetic strategies [9]. Furthermore, identifying differentially expressed genes (DEGs) and immune cell compositions can unearth potential biomarkers and therapeutic targets, thus enhancing the precision and efficacy of anesthetic management in cardiac surgeries.

While anesthesia’s impact on immediate perioperative outcomes is well-documented, a comprehensive understanding of its molecular and immunological effects at the genetic level in atrial tissues is lacking. Previous studies may have explored broader aspects of perioperative care, but the specific focus on anesthetic-induced gene expression changes and immune cell dynamics post-CABG surgery, as undertaken in this research, remains a research gap. By leveraging the GSE4386 dataset [10] and employing advanced analytical techniques, this study aims to bridge this gap and contribute novel insights to the existing body of knowledge.

The primary objectives of this research are to unravel the specific impact of sevoflurane and propofol on gene expression within atrial tissues post-CABG surgery and to delineate the associated changes in immune cell dynamics. The identification of DEGs, exploration of functional annotations and pathways through Gene Ontology (GO) and Kyoto Encyclopedia of Genes and Genomes (KEGG) analyses, and the characterization of immune cell compositions will collectively contribute to a nuanced understanding of the molecular and immunological responses to anesthetic agents in the context of cardiac surgeries. Ultimately, these findings aim to provide potential biomarkers and therapeutic targets, fostering advancements in anesthetic strategies for improved outcomes in CABG procedures.

## 2 Methods

### 2.1 Data acquisition and preprocessing

The expression profiling of GSE4386 produced by Lucchinetti et al., including 40 atrial samples, was downloaded from the National Center of Biotechnology Information Gene Expression Omnibus (http://www.ncbi.nlm.nih.gov/geo/), which was based on the Affymetrix Human Genome U133 Plus 2.0 Array platform. The 40 atrial samples were collected at the beginning and at the end of the off-pump CABG surgery and included 20 atrial samples from 10 patients receiving the anesthetic gas sevoflurane and 20 atrial samples from 10 patients receiving the intravenous anesthetic propofol. The sevoflurane and propofol were adjusted to maintain the blood pressure and heart rate within 20% of the baseline values [11]. In addition, patients with hemodynamic instability were excluded (sample GSM99282) [12]. Hierarchical clustering was employed to examine correlations and discrepancies among samples.

### 2.2 Gene expression analysis

To elucidate the distribution profiles of gene expression, box plots were generated post-outlier removal. These plots provided a visual representation of the variability in gene expression across all atrial tissue samples collected at the surgical termination point of CABG procedures.

DEGs between two groups, one exposed to the anesthetic gas sevoflurane and the other to intravenous anesthetic propofol, were identified using volcano plots. The Benjamini–Hochberg procedure [13] was used to adjust the raw *P*-values into false discovery rate. DEG selection criteria included an absolute value of log-fold change (|logFC|) of at least 0.58 and an associated *P*-value of less than 0.05.

### 2.3 Functional annotation and pathway analysis

The DEGs were further subjected to GO and KEGG enrichment analyses. Bubble plots illustrated the cellular component- (CC) and molecular function (MF)-related functional enrichments, while corresponding heatmaps displayed the distribution of DEGs across enriched categories. This step aimed to provide insights into the biological functions and pathways associated with the identified DEGs.

### 2.4 Friends analysis and correlation study

The Friends analysis approach assesses the functional correlation between different genes in a pathway, suggesting that a gene is more likely to be expressed if it interacts with other genes in the same pathway, and it is widely used to identify critical genes. To understand the relationships between genes, the top 20 DEGs based on |logFC| were analyzed using the Friends approach. A semantic similarity measure was employed, and visualized on a graph. Additionally, pairwise correlation analysis, specifically Spearman’s correlation, was executed on genes identified through Friends analysis.

### 2.5 Gene set enrichment analysis (GSEA)

GSEA was performed to investigate the enrichment of identified genes across different biological categories. Bubble plots were generated to illustrate gene enrichment in terms of CCs, MFs, and KEGG pathways.

### 2.6 Immune cell infiltration analysis

The cellular composition of atrial tissue samples was determined using various algorithms. The ESTIMATE algorithm calculated immune, stroma, and estimate scores, indicating cellular presence. MCPcounter and single sample gene set enrichment analysis (ssGSEA) algorithms provided insights into the abundance of specific immune cell types [14,15,16]. CIBERSORT algorithm [17] further characterized the composition and abundance of 22 distinct immune cell types within the atrial tissue samples.

## 3 Results

### 3.1 Preprocessing of the GSE4386 dataset

**Figure 1:**
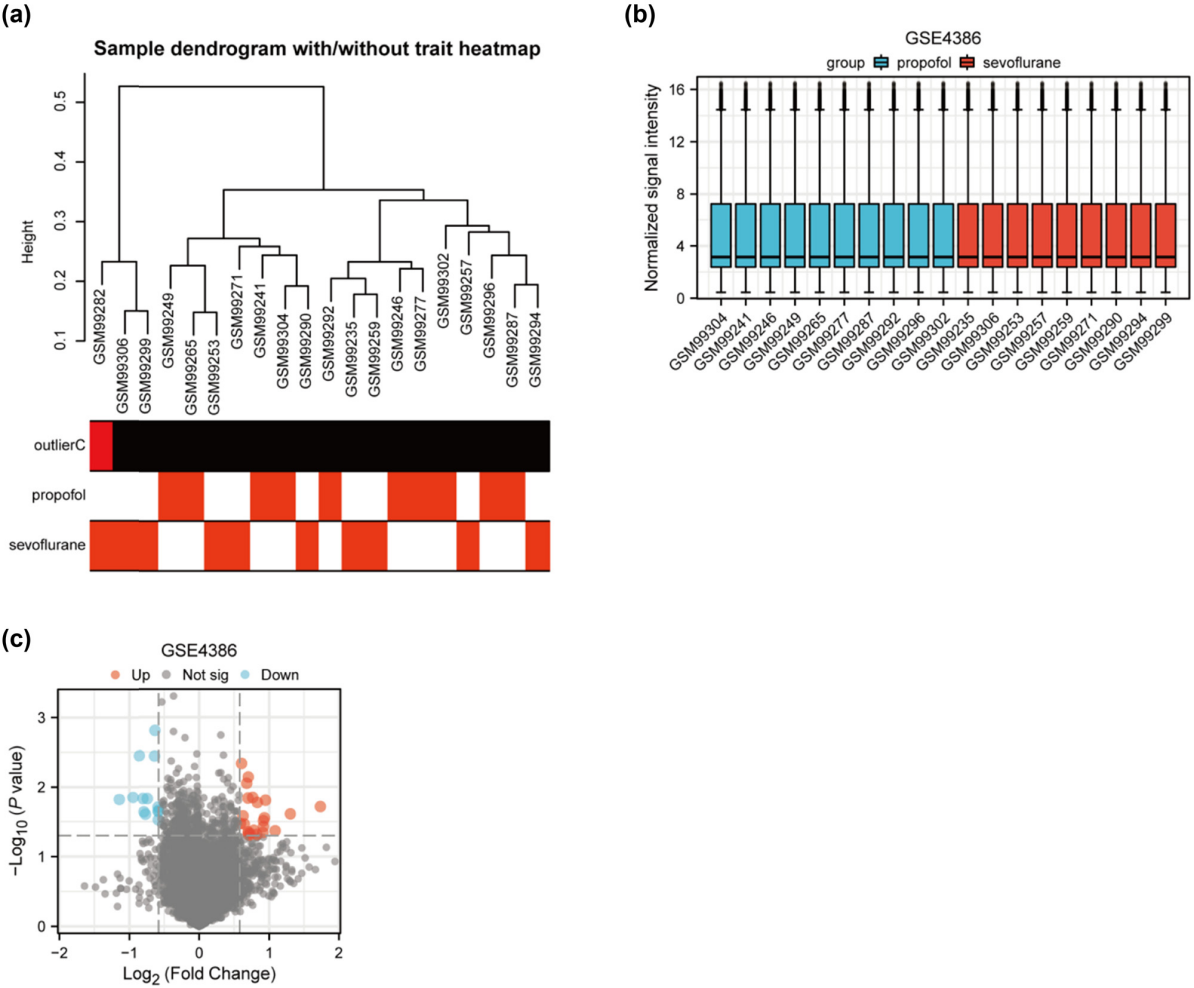
Preprocessing of the GSE4386 dataset. (a) Hierarchical clustering was applied to the GSE4386 dataset to elucidate correlations and discrepancies among samples. (b) Box plots were introduced to illustrate the varying distribution profiles of gene expression. (c) A volcano plot provides a comprehensive overview of the DEGs between two groups.

Hierarchical clustering was employed to reveal the correlations as well as discrepancies among the dataset’s samples (Figure 1a). Each sample is represented as a separate branch within the hierarchy. A distinct row-labeled “outlierC” has red bars indicating the outliers detected within the dataset. One notable outlier, specifically sample GSM99282, was marked as statistically significant and, thus, was removed from subsequent analyses to prevent data skewing. Following outlier removal, box plots were employed to visually represent the distribution profiles of gene expression across atrial tissue samples obtained during coronary artery bypass graft (CABG) surgery (Figure 1b). These samples were obtained at the surgical termination point of a procedure known as CABG. These box plots allow efficient visualization and comparison of data dispersion and symmetry for different categories of gene expression data. A volcano plot was constructed to comprehensively portray DEGs between two groups exposed to different anesthetic agents, sevoflurane and propofol (Figure 1c). One received the anesthetic gas sevoflurane and the other was subjected to an intravenous anesthetic propofol. The selection criteria for DEGs was based on the threshold of the absolute value of |logFC| being at least 0.58 and an associated *P*-value of less than 0.05. This critical threshold helps ascertain the genes with a significant degree of difference in their expression levels between the two aforementioned anesthetic groups. This analytical approach unveils molecular distinctions induced by distinct anesthetic interventions.

### 3.2 DEG representation through heatmap

**Figure 2:**
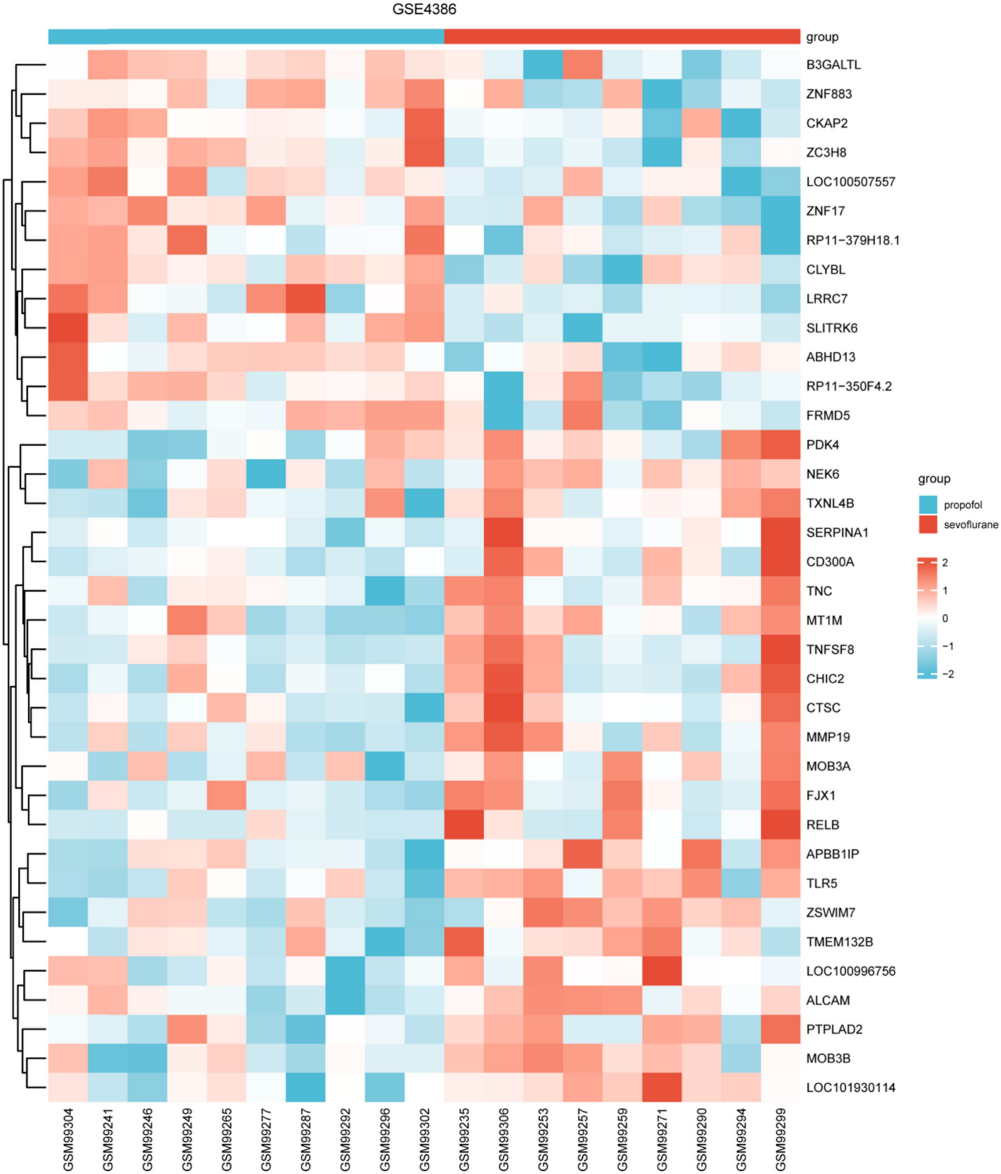
A heatmap representation of the DEGs between two comparison groups within the GSE4386 dataset. The heatmap is organized to visually depict the variance in gene expression levels, with each row representing a unique gene and each column symbolizing one sample. The color intensity within the heatmap correlates directly with the expression level of each gene: warmer tones (e.g., red) indicate higher expression, while cooler tones (e.g., blue) signify lower expression levels.

Figure 2 presents a heatmap representation elucidating the patterns of DEGs within the GSE4386 dataset. To facilitate comparison, row normalization has been applied, ensuring equitable representation of each gene’s expression levels across the samples. Furthermore, a meticulous clustering approach has been employed to organize the genes based on Euclidean distance. This visual depiction aids in recognizing gene expression patterns and facilitates a nuanced understanding of the molecular landscape between the compared groups. The sevoflurane group has a strong correlation with Pyruvate Dehydrogenase Kinase 4 (PDK4), Alpha-1-antiproteinase (SERPIN A1), CD300A, tumor necrosis factor (ligand) superfamily, member 8 (TNF SF8), and V-Rel Reticuloendotheliosis Viral Oncogene Homolog B (RELB); the propofol group has a strong correlation with SLIT and NTRK-like protein 6 (SLITRK6), leucine-rich repeat containing 7 (LRRC7), and abhydrolase domain containing 13 (ABHD13).

### 3.3 Friends approach and correlation study

**Figure 3:**
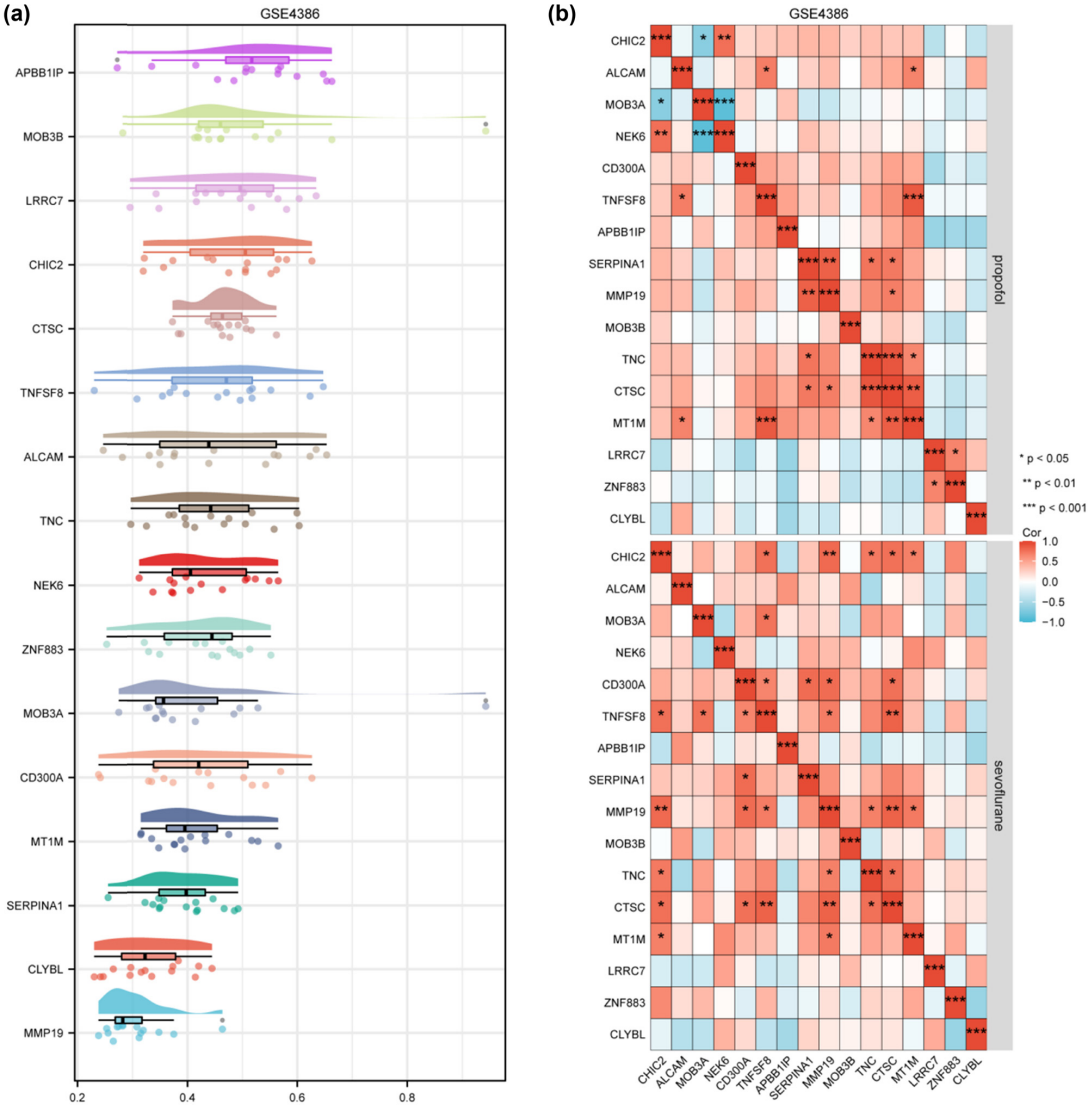
Analysis using the Friends approach and correlation study. (a) We investigated the top 20 genes selected based on the absolute value of |logFC| through Friends analysis. For visual interest, the *x*-axis displays the semantic similarity of a particular gene to all other genes, while the *y*-axis represents the corresponding genes themselves. (b) We executed a pairwise correlation analysis, specifically Spearman’s correlation, on the genes identified from the Friends analysis. The measure of statistical significance is denoted by asterisks, where *P < 0.05 shows a statistically significant correlation, **P < 0.01 indicates a highly significant correlation, and ***P < 0.001 represents a very highly significant correlation between pairwise genes.

The top 20 DEGs, identified through Friends analysis, were scrutinized to reveal their semantic similarity to other genes. This exploration, as depicted in Figure 3a, provides insights into the interconnectedness and functional relationships among genes.

### 3.4 Pairwise correlation analysis

Spearman’s correlation was executed on genes identified through Friends analysis, as presented in Figure 3b. This rigorous correlation study sheds light on potential co-regulated genes, enhancing our understanding of molecular interactions within the dataset.

### 3.5 GO and KEGG enrichment analysis of DEGs

**Figure 4:**
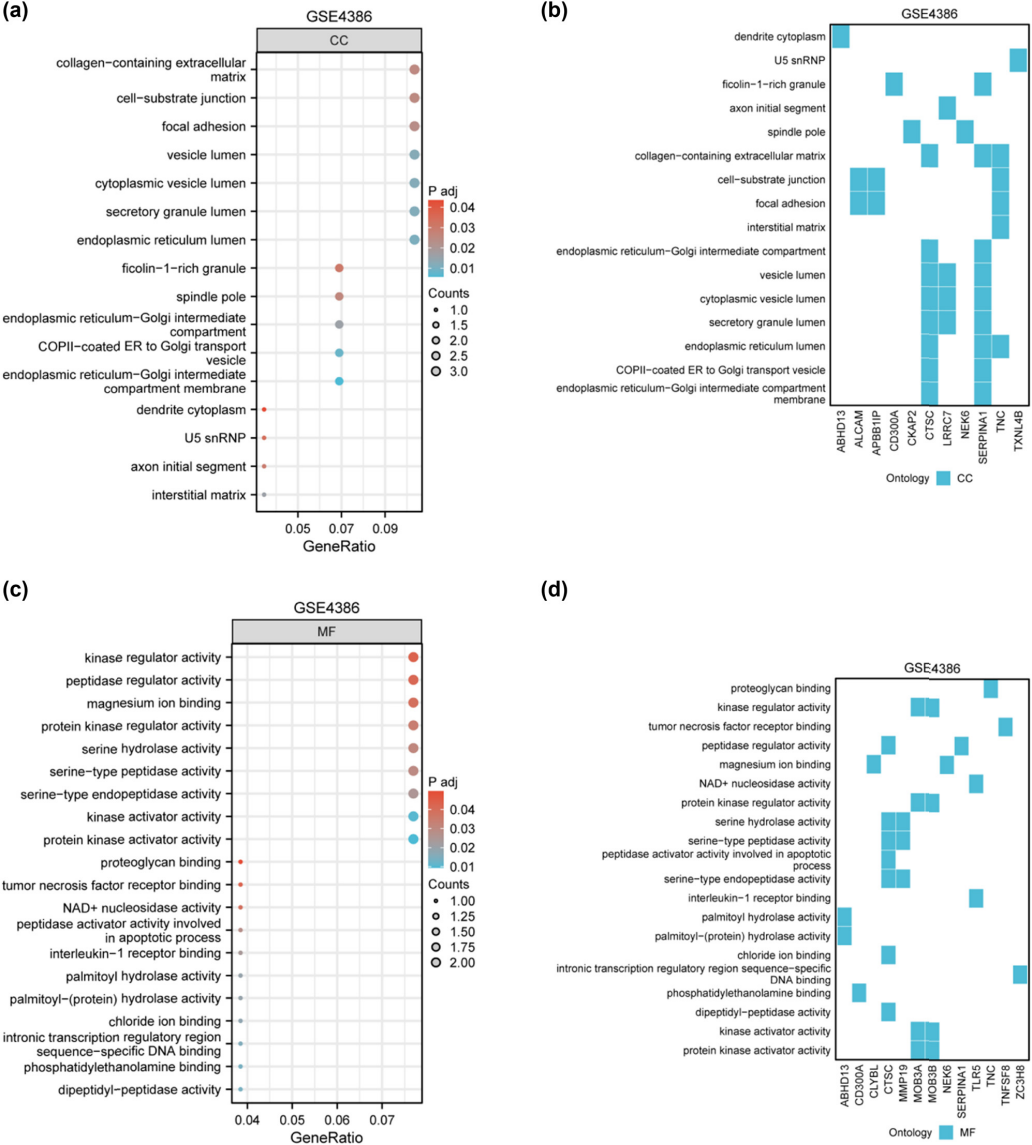
GO and KEGG enrichment analysis of DEGs. (a) Bubble plot illustrating CC-related functional enrichments of the DEGs. Each bubble in the plot corresponds to a specific CC and its size represents the count of DEGs related to that component. (b) Corresponding heatmap for the enrichments is depicted in (a), displaying the distribution of DEGs across the enriched categories. The *x*-axis represents genes in the selected items. (c) Bubble plot representing the enrichment of DEGs in terms of MF. Bubbles represent the specific molecular functional categories and their size correlates to the number of DEGs within that category. (d) The heatmap corresponding to Figure 4c, showing the distribution of DEGs in the enriched items or categories. The *x*-axis indicates genes in the chosen items.

Figure 4a illustrates a bubble plot highlighting the CC-related functional enrichments of DEGs. This analysis provides a detailed overview of cellular locations associated with the identified DEGs. Corresponding to Figure 4a, Figure 4b presents a heatmap showcasing the distribution of DEGs across enriched CC categories. This presentation enhances the clarity of DEG distribution within CCs. A bubble plot in Figure 4c delineates the enrichment of DEGs in terms of MF. This analysis delves into the functional roles of the identified DEGs. Figure 4d presents a heatmap corresponding to Figure 4c, depicting the distribution of DEGs across enriched MF categories. This comprehensive visualization aids in understanding the functional attributes of DEGs.

### 3.6 GSEA of identified genes

**Figure 5:**
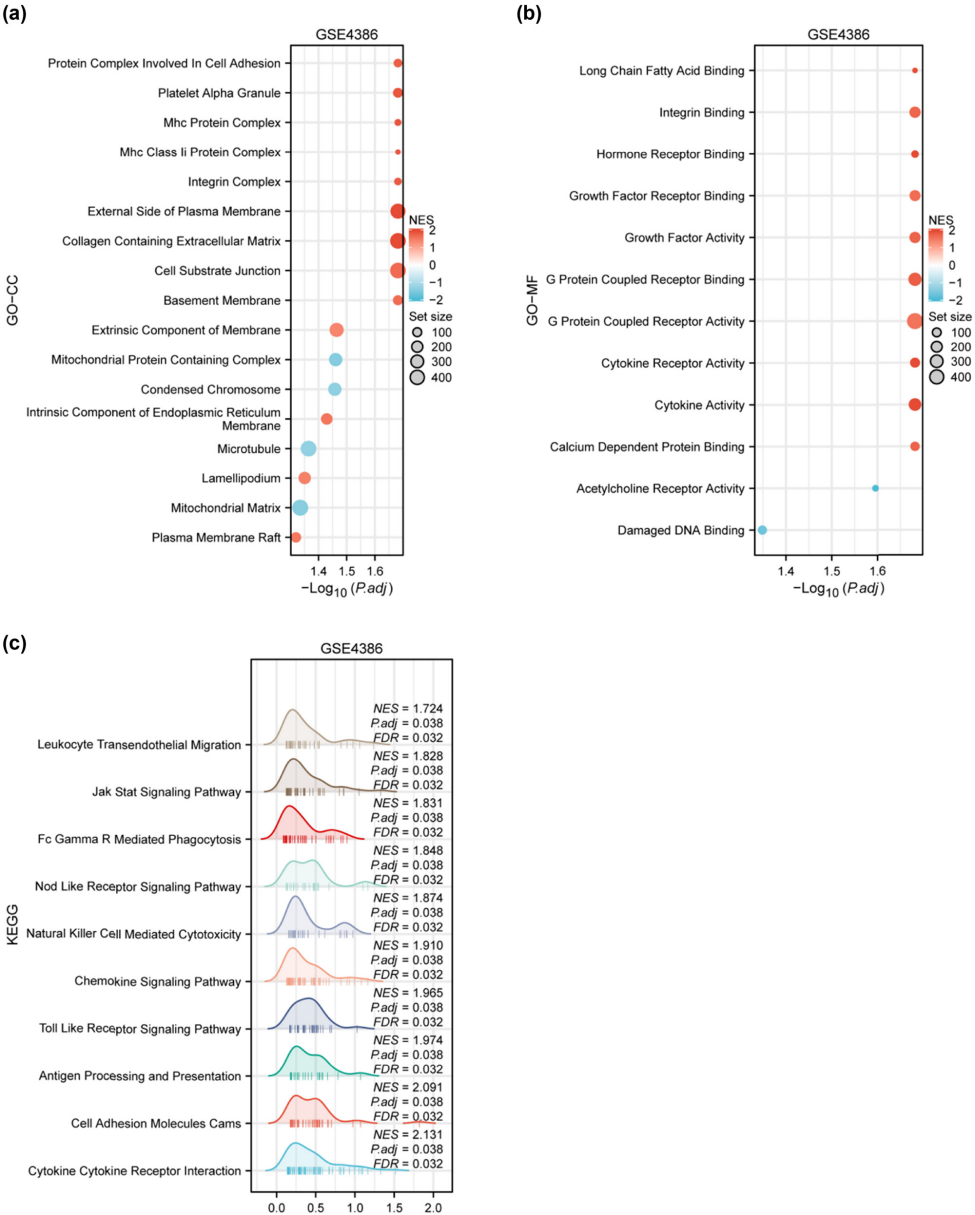
GSEA of identified genes in the GSE4386 dataset across different biological categories. (a) Bubble plot demonstrating the enrichment of genes within the category of CC. Each bubble signifies a distinct CC, with the size of the bubble representing the set size (number of molecules defined in the gene set) and the color indicating the normalized enrichment score (NES) after correction. (b) Bubble plot showcasing the enrichment of genes in terms of MF. This plot follows a similar format to (a), where each bubble corresponds to a specific MF. (c) Ridge plot illustrating the enrichment of genes across various KEGG pathways. The ridge plot provides a continuous curve representation for each enriched pathway, where the width of the curve along the *x*-axis indicates the density of genes associated with that pathway, and the *y*-axis positions represent different pathways.

Figure 5a and b, respectively, showcases bubble plots illustrating the enrichment of genes in CC, and MF. KEGG pathway enrichment analysis visualizes the top 9 pathways (Figure 5c), mainly involving Teukocyte Transendothelial Migration, Jak Stat Signaling Pathway Chemokine Signaling Pathway, Toll Like Receptor Signaling Pathway, and Cell Adhesion Molecules Cams. These visualizations provide a detailed overview of the functional implications and pathways associated with the identified genes in the GSE4386 dataset.

### 3.7 Immune cell infiltration in atrial tissue samples

**Figure 6:**
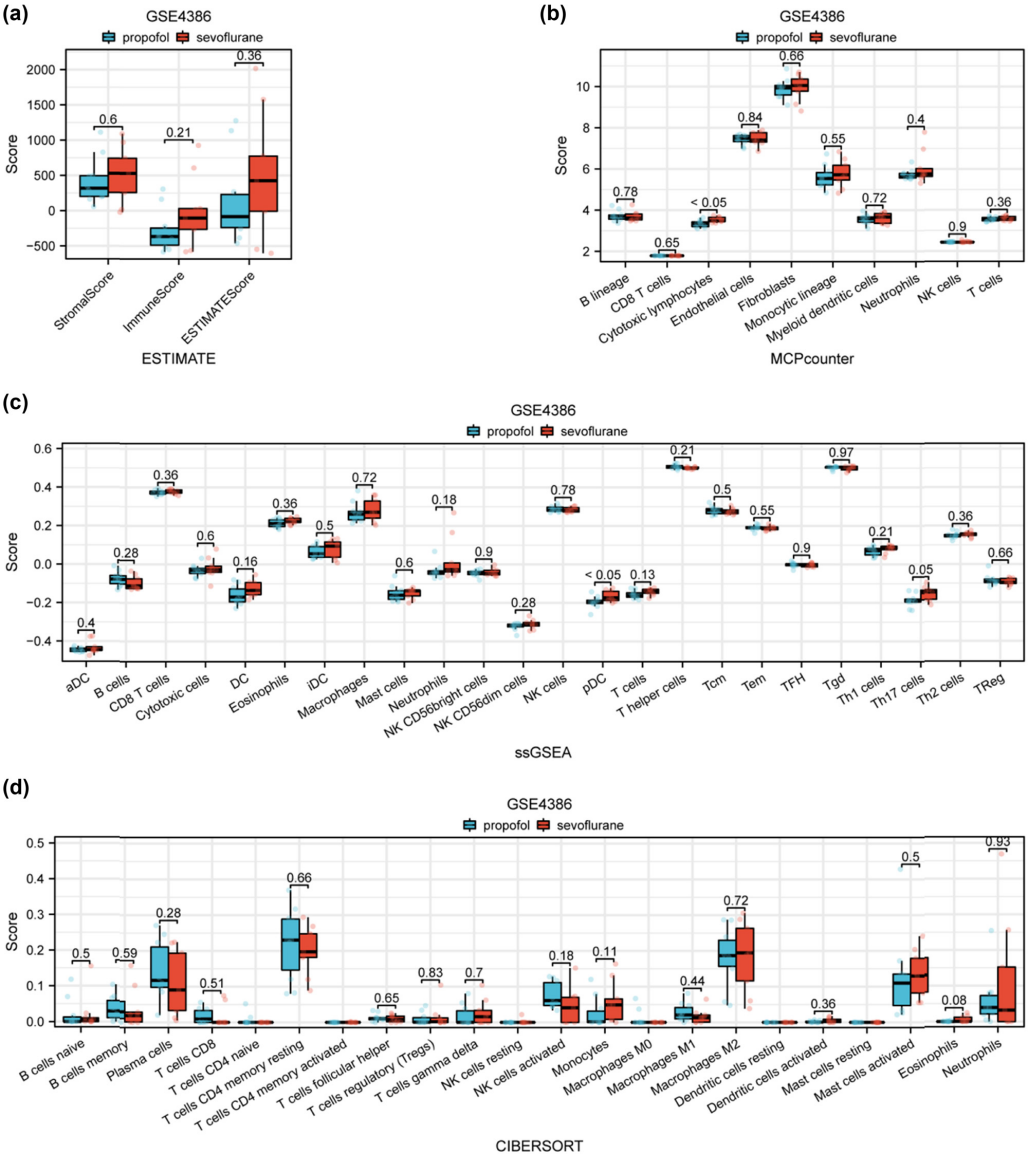
Immune cell infiltration in atrial tissue samples collected post-CABG surgery. (a) Immune, stroma, and estimate scores for atrial tissue samples calculated using the ESTIMATE algorithm, with higher scores indicating increased cellular presence. (b) Composition of immune cells within the atrial tissue samples as determined by MCPcounter algorithm. A noteworthy observation is the increased abundance of cytotoxic lymphocytes in the sevoflurane group as compared to the propofol group. (c) Landscape of immune cell composition within atrial tissue samples determined through the ssGSEA algorithm. (d) Composition and abundance of 22 distinct types of immune cells in the atrial tissue samples as inferred by the CIBERSORT algorithm.

Figure 6a displays immune, stroma, and estimate scores calculated using the ESTIMATE algorithm. Higher scores indicate increased cellular presence in atrial tissue samples post-CABG surgery. In Figure 6b, the composition of immune cells within atrial tissue samples is depicted using the MCPcounter algorithm. Notably, an increased abundance of cytotoxic lymphocytes is observed in the sevoflurane group compared to the propofol group. Figure 6c presents the landscape of immune cell composition within atrial tissue samples, determined through the ssGSEA algorithm. Plasmacytoid dendritic cells appear more abundant in the sevoflurane group compared to the propofol group. Figure 6d showcases the composition and abundance of 22 distinct types of immune cells in atrial tissue samples as inferred by the CIBERSORT algorithm. Among the findings, macrophage M0 and T cells CD4 naive are determined to be less prevalent in the sevoflurane group compared to the propofol group, providing valuable insights into immune cell dynamics in response to different anesthetic interventions.

## 4 Discussion

Compared with on-pump CABG, the levels of TNFα, heart-type fatty acid-binding protein, and creatine kinase-MB are significantly lower in off-pump CABG, suggesting a decreased systemic inflammatory response and reduced myocardial damage [18,19,20]. However, off-pump CABG surgery can also lead to ischemic injury. Several studies have identified that, to a certain extent, sevoflurane and propofol are effective cardioprotective anesthetic agents [21]. The exploration of anesthetic-induced gene expression changes and immune cell dynamics in atrial tissue post- CABG surgery, as elucidated by the analysis of the GSE4386 dataset, has yielded valuable insights into the intricate molecular and cellular responses to anesthetic agents, specifically sevoflurane and propofol [22,23]. This study contributes to the growing body of research aiming to understand the implications of anesthetics in the context of cardiac surgeries, shedding light on potential biomarkers and therapeutic targets.

The distinct expression patterns observed in the heatmaps and bubble plots between the sevoflurane and propofol groups underscore the influence of these anesthetic agents on the transcriptional landscape of atrial tissue post-CABG. The identification of DEGs through volcano plots, Friends approach, and Spearman’s correlation enhances our understanding of the specific genes that play crucial roles in mediating the effects of anesthetics. Correlation analyses provide a nuanced perspective, revealing not only individual gene changes but also how these changes interrelate, potentially uncovering key regulatory networks affected by sevoflurane and propofol.

The functional annotations and pathways revealed by GO and KEGG enrichment analyses further elucidate the biological processes influenced by anesthetic exposure. Reduce myocardial injury and inflammation after reperfusion injury through the JAK/STAT pathway. Chemokine-mediated monocyte infiltration into the damaged heart represents an initial step in inflammation during cardiac remodeling [24]. Toll-like receptors have been established to play an essential role in the activation of innate immunity by recognizing specific patterns of microbial components [25]. GSEA, by highlighting enriched biological categories, adds another layer of depth to our understanding of the broader functional implications of the observed gene expression changes. This knowledge is pivotal for deciphering the intricate mechanisms underlying anesthetic responses in cardiac tissues, potentially guiding future research and clinical interventions.

The immune cell infiltration analysis is a noteworthy component of this study, showcasing the complex dynamics of immune cells post-CABG. The differences in immune, stroma, and estimate scores, as demonstrated by the ESTIMATE algorithm, signify a potential role of anesthetics in shaping the microenvironment of the atrial tissue. The increased abundance of cytotoxic lymphocytes in the sevoflurane group, confirmed by ssGSEA and distinct immune cell compositions highlighted by the CIBERSORT algorithm, offers a comprehensive view of the immune landscape influenced by anesthetic exposure. CABG surgery can lead to ischemia/reperfusion injury, which is characterized by a strong inflammatory response. Interleukin (IL)-18, is a strong inflammatory mediator, that is released from cardiomyocytes and can be found in the systemic circulation of patients during and immediately after CABG surgery. The existing damage of endothelial glycocalyx in patients with ischemic heart disease is further impaired concurrently during the surgery due to the anesthesia-surgical technique used and intravascular fluid loading. This results in an increased incidence of adverse events, including myocardial infarction. IL-18 leads to the activation of lymphocyte cytotoxicity via cytotoxic mediators [26]. Notably, the prevalence of macrophage M0 is identified as significantly different between the sevoflurane and propofol groups, emphasizing the specific impact of each anesthetic agent on immune cell subpopulations. Sevoflurane group DEG CD300A is an inhibitory receptor that is expressed on various white blood cells, including mast cells and macrophages (MΦ). They are important cells in allergic inflammation [27].

Similar to studies, our findings align with prior research investigating the transcriptomic and immune responses to anesthetics in various contexts. For instance, studies exploring the impact of anesthetics on gene expression in other surgical scenarios have reported similar patterns of DEGs, emphasizing the need for personalized anesthetic strategies tailored to specific surgical procedures. Additionally, the observed alterations in immune cell compositions resonate with studies investigating immune responses in cardiac surgeries, highlighting the potential modulatory role of anesthetics in shaping these responses.

In conclusion, this comprehensive analysis significantly contributes to the understanding of anesthetic-induced gene expression changes and immune cell dynamics in atrial tissue post-CABG surgery. The identified DEGs and immune cell compositions not only enhance our knowledge of the molecular and cellular responses to sevoflurane and propofol but also offer potential biomarkers and therapeutic targets. Suggestions for future research directions can be based on the results of this study. These insights pave the way for further research aimed at refining anesthetic strategies in cardiac surgeries, ultimately improving patient outcomes and advancing personalized medicine in the field of anesthesia.

## 5 Limitations

However, the identified DEGs and pathways in this study were not investigated in animal models. Further study of this subject in animal models may be required in the future, simultaneously utilizing single-cell RNA sequencing technology for immune cell analysis.
